# Supplementary material for: Ca2+ channel and active zone protein abundance intersects with input-specific synapse organization to shape functional synaptic diversity
Source: eLife. 2024 Sep 18;12:RP88412. doi: 10.7554/eLife.88412 (PMC11410372; doi:10.7554/eLife.88412)
Supplement: Supplementary file 1. [file elife-88412-supp1.docx]

##### **Supplementary File 1a. Absolute values and statistics.** All comparisons, Ns (animals, NMJs, AZs (AZs), and statistical tests used in this study. All values are mean ± SEM.

| **Figure. Panel** | **Comparison** | **Ns** | **Values/Statistical tests** |
| --- | --- | --- | --- |
| Fig. 1C | Average P_r_ in type Ib and type Is | 6 animals, 6 NMJs | Ib Average P_r_ = 12.47 ± 0.88%  Is Average P_r_ = 25.69 ± 1.20%  Paired t test, p < 0.0001 |
| Fig. 1E | Cac^Td-Tomato-N^ to P_r_ correlation in type Is | 6 animals, 6 NMJs | Average type Is Pearson’s  r = 0.617 ± 0.028 |
| Fig. 1F | Cac^Td-Tomato-N^ to P_r_ correlation in type Ib | 6 animals, 6 NMJs | Average type Ib Pearson’s  r = 0.576 ± 0.035 |
| Fig. 1H | Cac^sfGFP-N^ levels at type Ib vs type Is | n = 12 animals,  36 NMJs | Type Ib mean: 1.000 ± 0.017  Type Is mean: 0.991 ± 0.028  t test, p = 0.78 |
| Fig. 1J | Slopes of Cac^Td-Tomato-N^ to P_r_ correlation at type Ib vs type Is | 6 animals, 6 NMJs  Type Ib: n = 531 AZs  Type Is: n = 365 AZs | Ib Slope = 0.085 ± 0.006  Is Slope = 0.154 ± 0.011  ANCOVA test, p < 0.0001 |
| Fig. 2F | Cac^HaloTag-N^ cluster area in type Ib and type Is | 5 animals, 14 NMJs  Type Ib: n = 80 boutons  5 animals, 19 NMJs  Type Is: n = 96 boutons | Type Ib = 0.0093 ± 0.0004 µm^2^  Type Is = 0.0098 ± 0.0004 µm^2^  Mann-Whitney test, p = 0.5906 |
| Fig. 2G | Cac^HaloTag-N^ localizations per cluster in type Ib and type Is | 5 animals, 14 NMJs  Type Ib: n = 80 boutons  5 animals, 19 NMJs  Type Is: n = 96 boutons | Type Ib = 69.39 ± 4.76  Type Is = 83.52 ± 5.67  Mann-Whitney test, p = 0.0928 |
| Fig. 2H | Cac^HaloTag-N^ cluster density in type Ib and type Is | 5 animals, 14 NMJs  Type Ib: n = 80 boutons  5 animals, 19 NMJs  Type Is: n = 96 boutons | Type Ib = 7185 ± 234.7 µm^-2^  Type Is = 8277 ± 342.6 µm^-2^  Mann-Whitney test, p = 0.0278 |
| Fig. 2I | Cac^HaloTag-N^ cluster density per NMJ in type Ib and type Is | Type Ib: n = 5 animals, 14 NMJs  Type Is: n = 5 animals 19 NMJs | Type Ib = 7300 ± 362.2 µm^-2^  Type Is = 8569 ± 584.0 µm^-2^  Wilcoxon test, p = 0.0076 |
| Fig. 3B | Brp levels at type Ib and type Is | n = 12 animals,  36 NMJs | Type Ib mean: 1.00 ± 0.02  Type Is mean: 0.79 ± 0.03  Unpaired T-test, p < 0.0001 |
| Fig. 3C | Cac^sfGFP-N^ :Brp ratio at type Ib vs type Is | n = 12 animals,  36 NMJs | Type Ib mean: 1.00 ± 0.01  Type Is mean: 1.30 ± 0.05  Paired T-test, p < 0.0001 |
| Fig. 3D | Cac^sfGFP-N^ :Brp correlation | 12 animals, 36 NMJs  Ib: n = 5349 AZs  Is: n = 2625 AZs | Type Ib slope: 0.874 ± 0.005  Type Is slope: 0.787 ± 0.009  ANCOVA, p < 0.0001 |
| Fig. 3G | Cac^sfGFP-N^ levels in type Ib vs Is in Control and *brp^-/-^* | 9 animals  Control Ib: n = 29 NMJs  *brp^-/-^* Ib: n = 31 NMJs  Control Is: n = 29 NMJs  *brp^-/-^* Is: n = 31 NMJs | Control Ib mean = 1.00 ± 0.03  *brp^-/-^* Ib mean = 0.33 ± 0.01; p < 0.0001  Control Is mean = 1.00 ± 0.03  *brp^-/-^* Is mean = 0.45 ± 0.02; p < 0.0001  Kruskal-Wallis test adjusted p values vs Control |
| Fig. 3H | *brp^-/-^*:Control ratio of Cac^sfGFP-N^ levels in type Ib vs Is | n = 9 animals,  31 NMJs | Ib ratio = 0.334 ± 0.010  Is ratio = 0.451 ± 0.018  Mann-Whitney test, p < 0.0001 |
| Fig. 4C | Brp levels at *cac^sfGFP-N^* type Ib and type Is terminals in control vs PhTx | Control: n = 12 animals, 40 NMJs  PhTx: n = 12 animals, 40 NMJs | Control Ib = 1.000 ± 0.047  PhTx Ib = 1.488 ± 0.068; p < 0.0001  Control Is = 1.000 ± 0.050  PhTx Is = 1.493 ± 0.076; p < 0.0001  Kruskal-Wallis test adjusted p values vs Control |
| Fig. 4D | Cac^sfGFP-N^ levels at *cac^sfGFP-N^* type Ib and type Is terminals in control vs PhTx | Control: n = 12 animals, 40 NMJs  PhTx: n = 12 animals, 40 NMJs | Control Ib = 1.000 ± 0.044  PhTx Ib = 1.325 ± 0.059; p < 0.0001  Control Is = 1.000 ± 0.048  PhTx Is = 1.300 ± 0.058; p = 0.0002  ANOVA test adjusted p values vs Control |
| Fig. 4G | Cac^sfGFP-N^ levels at *cac^sfGFP-N^*; *brp^-/-^* type Ib and type Is terminals in control vs PhTx | Control: n = 12 animals, 43 NMJs  PhTx: n = 12 animals, 43 NMJs | Control Ib = 1.000 ± 0.041  PhTx Ib = 1.029 ± 0.038; p = 0.8475  Control Is = 1.000 ± 0.046  PhTx Is = 0.8288 ± 0.035; p = 0.0058  ANOVA test adjusted p values vs Control |
| Fig. 5C | EJPs of endogenously tagged subunits | WT: n = 9 NMJs  Stj: n = 8 NMJs  Stolid: n = 11 NMJs  Ca-β: n = 9 NMJs | WT mean = 30.21 ± 4.09 mV  Stj mean = 41.17 ± 4.32 mV; p = 0.14  Stolid mean = 26.44 ± 2.82 mV; p = 0.80  Ca-β mean = 36.49 ± 4.09 mV; p = 0.52  ANOVA, adjusted p values vs WT |
| Fig. 5D | mEJPs of endogenously tagged subunits | WT: n = 9 NMJs  Stj: n = 8 NMJs  Stolid: n = 11 NMJs  Ca-β: n = 9 NMJs | WT mean = 1.02 ± 0.08 mV  Stj mean = 1.12 ± 0.07 mV; p = 0.92  Stolid mean = 1.11 ± 0.09 mV; p = 0.99  Ca-β mean = 1.16 ± 0.09 mV; p = 0.57  Kruskal-Wallis test adjusted p values vs WT |
| Fig. 5E | QC of endogenously tagged subunits | WT: n = 9 NMJs  Stj: n = 8 NMJs  Stolid: n = 11 NMJs  Ca-β: n = 9 NMJs | WT mean = 29.63 ± 2.96  Stj mean = 37.02 ± 3.93; p = 0.34  Stolid mean = 24.73 ± 2.63; p = 0.60  Ca-β mean = 32.79 ± 4.34; p = 0.86  ANOVA, adjusted p values vs WT |
| Fig. 6E | Ca-β^V5-C^ levels at type Ib vs type Is | n = 11 animals,  27 NMJs | Type Ib mean: 1.00 ± 0.04  Type Is mean: 0.90 ± 0.04  Mann-Whitney Test, p = 0.06 |
| Fig. 6F | Stj^V5-N^ levels at type Ib vs type Is | n = 9 animals,  28 NMJs | Type Ib mean: 1.00 ± 0.05  Type Is mean: 0.76 ± 0.05  Unpaired t test, p = 0.0008 |
| Fig. 6G, H | Cac^sfGFP-N^ :Stj^V5-N^ correlation | 5 animals, 22 NMJs  Ib: n = 4012 AZs  Is: n = 2211 AZs | Type Ib slope: 0.912 ± 0.009  Type Is slope: 0.914 ± 0.012  ANCOVA, p = 0.90 |
| Fig. 7C | Stj^V5-N^ levels at type Ib and type Is terminals in control vs PhTx | Control: n = 6 animals, 34 NMJs  PhTx: n = 6 animals, 26 NMJs | Control Ib = 1.00 ± 0.01  PhTx Ib = 1.27 ± 0.03; p < 0.0001  Control Is = 1.00 ± 0.02  PhTx Is = 1.26 ± 0.02; p < 0.0001  Kruskal-Wallis test adjusted p values vs Control |
| Fig. 7F | Stj^V5-N^ levels at type Ib and type Is terminals in control vs *GluRIIA^-/-^* | Control: n = 7 animals, 37 NMJs  *GluRIIA^-/-^*: n = 7 animals, 26 NMJs | Control Ib = 1.00 ± 0.02  *GluRIIA^-/-^* Ib = 1.18 ± 0.03; p < 0.0001  Control Is = 1.00 ± 0.02  *GluRIIA^-/-^* Is = 1.17 ± 0.03; p < 0.0001  ANOVA test adjusted p values vs Control |
| Fig. S1D | EJPs of Cac endogenous tags | Control: n = 11 NMJs  *cac^HaloTag-N^*: n = 8 NMJs  *cac^Td-Tomato-N^*: n = 8 NMJs | Control mean = 42.51 ± 2.08 mV  *cac^HaloTag-N^* mean = 43.02 ± 1.92 mV; p = >0.99  *cac^Td-Tomato-N^* mean = 40.07 ± 0.99 mV; p = 0.25  Kruskal-Wallis test adjusted p values vs Control |
| Fig. S1E | mEJPs of Cac endogenous tags | Control: n = 11 NMJs  *cac^HaloTag-N^*: n = 8 NMJs  *cac^Td-Tomato-N^*: n = 8 NMJs | Control mean = 0.88 ± 0.07 mV  *cac^HaloTag-N^* mean = 0.77 ± 0.07 mV; p = 0.47  *cac^Td-Tomato-N^* mean = 0.93 ± 0.06 mV; p = 0.86  ANOVA, adjusted p values vs Control |
| Fig. S1F | QC of Cac endogenous tags | Control: n = 11 NMJs  *cac^HaloTag-N^*: n = 8 NMJs  *cac^Td-Tomato-N^*: n = 8 NMJs | Control mean = 49.97 ± 2.56 mV  *cac^HaloTag-N^* mean = 59.83 ± 6.76 mV; p = 0.23  *cac^Td-Tomato-N^* mean = 44.34 ± 2.95 mV; p = 0.60  ANOVA, adjusted p values vs Control |

**Supplementary File 1b. Imaging details.** This table contains detailed information on how each protein was labeled and visualized using live and fixed confocal microscopy and STORM imaging. All secondary antibodies were incubated at RT for 2 hours at a concentration of 1:500.

| **Imaging Method** | **Protein** | **Genetic line** | **Imaging Reagents** | **Sample processing** |
| --- | --- | --- | --- | --- |
| Confocal | Cac | *cac^sfGFP-N^* (Gratz et al., 2019) | anti-GFP AF488 conjugate (ThermoFisher- RRID:AB_221477) | Fix: Bouins or 4% PFA  Staining: 1:500, 2 hours at RT |
| Confocal | Stj | *stj^V5-N^* ( this study) | *Primary:* anti-V5 monoclonal (ThermoFisher- RRID:AB_2556564)  *Secondary:* Goat anti-Mouse IgG Highly Cross-Adsorbed AF488 (ThermoFisher- RRID:AB_2534088) | Fix: Methanol  Primary staining: 1:500, overnight at 4°C  *Note, best in 488 channel |
| Confocal | Stolid | *stolid^V5-N^* ( this study) | *Primary:* anti-V5 monoclonal (ThermoFisher- RRID:AB_2556564)  *Secondary:* Goat anti-Mouse IgG Highly Cross-Adsorbed AF488 (ThermoFisher- RRID:AB_2534088) | Fix: Bouins  Primary staining: 1:500, overnight at 4°C |
| Confocal | Ca-β | *Ca-β^V5-C^* (this study) | *Primary:* anti-V5 monoclonal (ThermoFisher- RRID:AB_2556564)  *Secondary:* Goat anti-Mouse IgG Highly Cross-Adsorbed AF488 (ThermoFisher- RRID:AB_2534088) | Fix: Bouins  Primary staining: 1:500, overnight at 4°C |
| Confocal | Brp |  | *Primary:* anti-Brp *(*DSHB- RRID:AB_2314866)  *Secondary:* Goat anti-Mouse IgG Highly Cross-Adsorbed AF568 (ThermoFisher- RRID:AB_144696 ) | Fix: Bouins or Methanol  Primary staining: 1:100 overnight at 4°C |
| Confocal | HRP |  | anti-HRP AF488 conjugate (Jackson ImmunoResearch- RRID:AB_2338965),  anti-HRP AF647 conjugate (Jackson ImmunoResearch- RRID:AB_2338967) | Fix: Bouins or Methanol  Primary staining: 1:500, 2 hours at RT |
| Live  Imaging | Cac | *cac^Td-Tomato-N^* (this study) |  |  |
| STORM | Cac | *cac^HaloTag-N^* (this study) | JaneliaFluor 646 HaloTag Ligand (Promega #GA1120) | Live label: 500nM for 20 min in dark box at RT  Fix: 4% PFA for 30 min at RT in dark box |
