# Supplementary material for: Ca2+ channel and active zone protein abundance intersects with input-specific synapse organization to shape functional synaptic diversity
Source: eLife. 2024 Sep 18;12:RP88412. doi: 10.7554/eLife.88412 (PMC11410372; doi:10.7554/eLife.88412)
Supplement: Transparent reporting form [file elife-88412-transrepform1.pdf]

eLIFE
1st Floor
24 Hills Road
Cambridge CB2 1JP, UK
P 01223 855340
W elifesciences.org
T @elife

eLife's transparent reporting form

We encourage authors to provide detailed information *within their submission* to facilitate the interpretation and replication of experiments. If you have any questions, please contact us: editorial@elifesciences.org.

Sample-size estimation

- You should state whether an appropriate sample size was computed when the study was being designed
- You should state the statistical method of sample size computation and any required assumptions
- If no explicit power analysis was used, you should describe how you decided what sample (replicate) size (number) to use

Please outline where this information can be found within the submission (e.g., page numbers or figure legends), or explain why this information doesn't apply to your submission:

Sample-size for each experiment was determined based on previously published studies. Fixed-tissue imaging experiments included at least 5 biological replicates (animals) and a range of 14-40 neuromuscular junctions (NMJs) depending on experiment. Functional imaging and electrophysiological experiments include at least 4 biological replicates (animals) and a range of 6-11 NMJs. Sample size for each experiment (animals and NMJs) is reported in Table S1.

Replicates

- You should report how often each experiment was performed
- You should include a definition of biological versus technical replication
- The data obtained should be provided and sufficient information should be provided to indicate the number of independent biological and/or technical replicates
- If you encountered any outliers, you should describe how these were handled
- Criteria for exclusion/inclusion of data should be clearly stated
- High-throughput sequence data should be uploaded before submission, with a private link for reviewers provided (these are available from both GEO and ArrayExpress)

Please outline where this information can be found within the submission (e.g., page numbers or figure legends), or explain why this information doesn't apply to your submission:

eLife Sciences Publications, Ltd is a limited liability non-profit non-stock corporation incorporated in the State of Delaware, USA, with company number 5030732, and is registered in the UK with company number FC030576 and branch number BR015634 at the address 1st Floor, 24 Hills Road, Cambridge CB2 1JP | August 2014

For all experiments, biological replicate is defined as the number of animals used for testing. Technical replication is defined as the number of times an experiment including at least 5 biological replicates was performed. For confocal fixed imaging studies comparing type Ib and type Is inputs, images were collected and analyzed only if they contained both neuronal subtypes within the same image. For functional imaging studies, NMJs were included only if they contained both type Ib and type Is inputs and stimulation was able to recruit both. For electrophysiology, only cells with resting membrane potential of -60 to -80 mV and resistance of ≥ 5 MΩ were included.

Statistical reporting

- Statistical analysis methods should be described and justified
- Raw data should be presented in figures whenever informative to do so (typically when N per group is less than 10)
- For each experiment, you should identify the statistical tests used, exact values of N, definitions of center, methods of multiple test correction, and dispersion and precision measures (e.g., mean, median, SD, SEM, confidence intervals; and, for the major substantive results, a measure of effect size (e.g., Pearson's r, Cohen's d)
- Report exact p-values wherever possible alongside the summary statistics and 95% confidence intervals. These should be reported for all key questions and not only when the p-value is less than 0.05.

Please outline where this information can be found within the submission (e.g., page numbers or figure legends), or explain why this information doesn't apply to your submission:

Statistical analysis was performed as detailed in Materials and Methods *Experimental Design and statistical analysis* section. All values for *N* (animals and NMJs), mean, SEM, and results of statistical tests (p values or Pearson's r) for each figure panel are reported in Table S1. All data generated, descriptive statistics, and statistical tests run for each experiment are included in the source data files associated with each figure. Data are presented as dot plots to maximize transparency.

(For large datasets, or papers with a very large number of statistical tests, you may upload a single table file with tests, Ns, etc., with reference to page numbers in the manuscript.)

Additional data files ("source data")

- We encourage you to upload relevant additional data files, such as numerical data that are represented as a graph in a figure, or as a summary table
- Where provided, these should be in the most useful format, and they can be uploaded as "Source data" files linked to a main figure or table

- Include model definition files including the full list of parameters used
- Include code used for data analysis (e.g., R, MatLab)
- Avoid stating that data files are "available upon request"

Please indicate the figures or tables for which source data files have been provided:

Source data is included in a "Source Data" file associated with each figure. Custom code used to analyze STORM data is also provided in ipynb format. All source data, including statistical tests and raw images, can also be found in the following Harvard Dataverse dataset https://doi.org/10.7910/DVN/GGP3UM.
